# Ecological information and approaches needed for risk communication dialogs for acute or chronic environmental crises

**DOI:** 10.1111/risa.13940

**Published:** 2022-05-01

**Authors:** Joanna Burger

**Affiliations:** 1 Cell Biology and Neurosciences, NIEHS Center of Excellence, Environmental and Occupational Health Sciences Institute (EOHSI), Ecology and Evolution Graduate Program, and Pinelands Research Station, Rutgers University, Piscataway, New Jersey, USA; 2 Consortium for Risk Evaluation with Stakeholder Participation (CRESP), Rutgers University, Piscataway, New Jersey, USA

**Keywords:** eco-cultural, eco-receptors, environmental justice, risk communication

## Abstract

Scientists, social scientists, risk communicators, and many others are often thrust into a crisis situation where they need to interact with a range of stakeholders, including governmental personnel (tribal, U.S. federal, state, local), local residents, and other publics, as well as other scientists and other risk communicators in situations where information is incomplete and evolving. This paper provides: (1) an overall framework for thinking about communication during crises, from acute to chronic, and local to widespread, (2) a template for the types of ecological information needed to address public and environmental concerns, and (3) examples to illustrate how this information will aid risk communicators. The main goal is providing an approach to the knowledge needed by communicators to address the challenges of protecting ecological resources during an environmental crisis, or for an on-going, chronic environmental issue. To understand the risk to these ecological resources, it is important to identify the type of event, whether it is acute or chronic (or some combination of these), what receptors are at risk, and what stressors are involved (natural, biological, chemical, radiological). For ecological resources, the key information a communicator needs for a crisis is whether any of the following are present: threatened or endangered species, species of special concern, species groups of concern (e.g., neotropical bird migrants, breeding frogs in vernal ponds, rare plant assemblages), unique or rare habitats, species of commercial and recreational interest, and species/habitats of especial interest for medicinal, cultural, or religious activities. Communication among stakeholders is complicated with respect to risk to ecological receptors because of differences in trust, credibility, empathy, perceptions, world view valuation of the resources, and in many cases, a history of misinformation, disinformation, or no information. Exposure of salmon spawning in the Columbia River to hexavalent chromium from the Hanford Site is used as an example of communication challenges with different stakeholders, including Native Americans with Tribal Treaty rights to the land.

## 1 | INTRODUCTION

Risk communication is difficult under ordinary circumstances, but becomes more complex in a crisis, or in a chronic exposure scenario that eludes management. Crises can be caused by natural, biological, and nuclear and chemical stressors or exposures. They may operate on different temporal and spatial scales, impacting a variety of receptors, or vary in importance to different stakeholders. While risk communication has often been examined with respect to the stressor (e.g., chemical or nuclear accident), or to the people at risk (physical or emotional health), many crises have equal or more devastating results on the surrounding ecosystems. Think, for example of recent environmental crises such as Chernobyl (Davydchuk, 1997), Fukushima (Denning & Mubayi, 2017; Kusumi et al., 2017), the Exxon Valdez (Lance et al., 2001), Deepwater Horizon Oil Spill (McNutt et al., 2012; Montevecchi et al., 2012), or Hurricane Katrina (McKee & Cherry, 2009). While each of these “disasters” had devastating human health and cultural effects, they also had enormous direct effects on species, ecological communities, and ecosystems. Some species died or declined precipitously around these accidents, in some cases populations are still depressed decades later, and the landscapes have not recovered (Garnier-Laplace et al., 2015; Peterson et al., 2003). On the other hand, the long-term effects of a severe disaster can sometimes result in different or contradictory effects (Moller & Mousseau, 2006). Credible and science-based information is needed and is essential for successful communication and dialog, not only for human health but for ecological and environmental health as well.

In the cases of hurricanes and accidents, the effects on both people and ecosystems are direct and evident—death and destruction. However other crises also have effects on ecoreceptors and ecosystems, including crises normally thought of as only human such as the COVID-19 pandemic (Kurzweil et al., 2021). But these too affect ecological systems, if only through a change in human behavior in those systems. Indirect effects were most obvious during the COVID-19 pandemic when people all over the world were isolating and staying indoors (thus providing less human disturbance), in other places people were flocking to wild habitats (creating more disturbance), and in still others the pandemic led to increased deforestation for gardens, and an increased use of bushmeat (subsistence reliance on wild animals) (Heinrichs, 2020; Rutz et al., 2020). Ecologists and conservationists might be the most directly concerned with the adverse effects on species and ecosystems during an environmental crises, but many other people are concerned as well, including governmental agencies, natural resource managers and regulators, conservation organizations, consumptive and nonconsumptive resource users, the general public, and a range of people interested in the environment for aesthetic, cultural, religious, and existence values (Chan et al., 2012; Davidson, 2013). At the least, providing clean air and water are critical (Costanza et al., 2017). Whether the environmental stressor is an acute crisis or chronic, some basic information about the risk to ecological systems is useful, timely, and complements examinations of the injuries to people.

This paper briefly (1) provides a conceptual model for the information needed by risk communicators for a crisis, (2) examines the basic ecological information needed before communicating the potential hazards and injuries, (3) gives some examples where understanding ecological and eco-cultural resources is essential, and finishes with (4) some important lessons derived from the examples for communicators who might be faced with a crisis or chronic environmental problem that has chronic exposures and risks, This information is essential so that communicators can provide important and accurate information to the public, agencies, or others during a crisis, and it provides a framework for understanding risk to the systems that humans rely on for food, fiber, clean air and water, and a full range of cultural and religious purposes (Bohnee et al., 2011; Burger, 2008; Burger et al., 2022). While imparting ecological information, it is still critical to consider the following questions: What is my communication for? What do I want to accomplish? What does my audience want to accomplish? What facts or risks do I want to impart? Will facts heal or solve the problem? Do I have solutions, as well as problems? And are the solutions equitable and just?

## 2 | ECOLOGICAL INFORMATION NEEDED FOR COMMUNICATING RISK TO AN ECOSYSTEM IN A CRISES

To understand the potential risk to species and ecosystems it is first important to understand the context of the event, be it natural or anthropogenic, acute or chronic, or driven by an accident or not, as well as by the type of stressor (e.g., fire and smoke, chemical, radionuclides, virus). Events act through potential sources of exposures, which can result in acute crises, or chronic crises. An acute event becomes chronic, for example in the case of an oil spill. Moreover, sometimes a long-standing chronic exposure suddenly becomes a crisis because the information suddenly becomes public and attracts regional or national attention (e.g., drinking water in Newark New Jersey or Flint, Michigan, Zahran et al., 2017). Different types of events can lead to an ecological risk crisis, and these events can be natural or anthropogenic, or some combination thereof. Other factors include the sources of exposure, the spatial and temporal scope of the exposure, and the range of ecological effects. As a basis for risk communication, the communicator should know what information people already possess, why they are interested, and what they need. The severity of consequences and effects varies depending on each level of information (causes, events, sources, and exposures) that leads to exposure and risk for ecological resources. The consequences or effects obviously depend upon causes, events, sources, and exposures. The communication challenges relate to having scientifically credible, up-to-date information.

**FIGURE 1:**
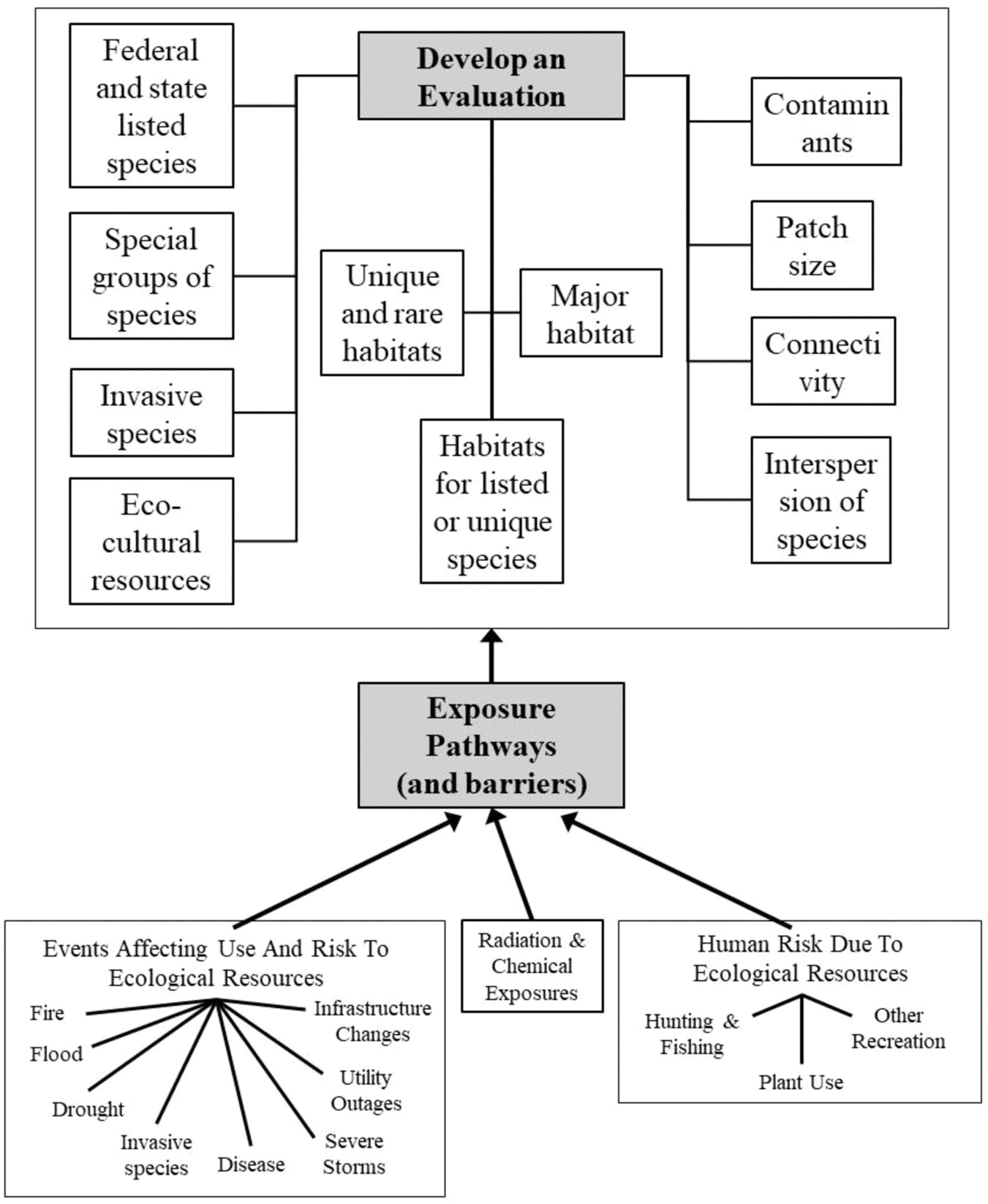
Kinds of receptors and issues to consider when communicating with governmental agencies, groups of concerned stakeholders, and the public

Information challenges (or needs) include immediately identifying what species or species groups are at risk, what populations or communities are at risk, what unique habitats are at risk, and what eco-cultural resources and services are at risk. The major ecological endpoints that must be considered are federal and state-listed species, special species groups, and unique and rare habitats, but equally important are invasive species, major or common habitats at risk regionally, and landscape features, such as patch size, connectivity among habitat types, and the interspersion of habitats (Figure 1).

Partly the risk to ecological resources depends upon whether there are barriers to exposure. The types of barriers that can prevent injuries to ecological resources differ depending upon the causes and events. For example, a wide river (a natural barrier) may prevent a fire from reaching a rare or unique habitat, a cap (engineered barrier) may prevent waste from seeping into another rare or even a common habitat. These barriers can be incorporated in any conceptual site mode (CSM). Further, the public will likely want to know the temporal and spatial scales and patterns of exposure. While the spatial extent may be possible to describe at any one time, it is often not possible to predict the temporal trends of ecological injuries, or the time to recovery.

Ideally, risk communicators need to be aware of all of these issues for their local environments or have ready access to ecologists on their staffs. Identification of ecologists, as well as others who might provide information about the type and nature of a crisis (or chronic adverse exposures) is one of the first tasks of a communicator because trying to find experts during an acute crisis is much more difficult than leisurely assembling a file of people to provide up-to-date, credible, scientifically based information and data. Similarly, the legal status of species and of habitats can change over time, and periodically risk communicators should check these changes, or know exactly where to find any updated information.

The kinds of ecological information described above for any crisis, or in a chronic environmental exposure are summarized in Table 1. This table provides an overview of some of the questions and concerns for each of the major ecological endpoints, as well as communication challenges and information needs. At its simplest level, it is a matter of determining the appropriate indicators of adverse effects, or that require examination. The first level of examination is whether there are federal- or state-listed endangered or threatened species; these species have already been evaluated as species requiring attention and are of importance in ecological systems. Thus, having available lists of these species (or knowing how to acquire them quickly) is essential for anyone required to communicate the risk to ecological resources because of a crisis or emergency. An additional first level of concern is whether there are unique or rare habitats that should be protected. Examples include: (1) vernal ponds that might house important groups of frogs or salamanders during the breeding season, (2) habitats with assemblages of nesting colonial waterbirds that should be protected from an oil spill, (3) fish spawning habitats where a significant percent of the population breeds, or (4) rare caves, cliffs, or other unusual landscape features. Habitats of concern also include cultural resources for tribes, subsistence communities, or other ethnic or religious group.

The questions and concerns deal not only with species and habitats of ecological structure and function, but the importance of these resources to people of all different groups and cultures. That is, ecological resources do not only provide goods and services, but a range of ecocultural aspects of cultural, religious, and aesthetic viewpoints. More detailed information on what kinds of information is necessary to evaluate the risk to ecological resources, particularly after disruptions, can be found in Burger (2019) and Burger et al. (2020, 2022).

The traditional indices or endpoints for ecological risk are far narrower than the complexities in Table 1, but provide an initial understanding of the issues involved (Table 2). These detailed endpoints will likely not be considered when a crisis is first identified, but are important for communicating about the long-term effects, restoration potential, recovery time, and when people can again use the resources and habitats. This information will require trained ecologists, and the information is most likely to be available for parks, forests, and other ecological reserves that have existing staffs. However, this information is not always readily available even for some federal or state lands (i.e. see chapter about J. Salisbury for forest fire), for particular crises with ecological consequences (i.e. see chapter about K. Berlin for climate change, and C. Safina for fisheries), or for ecological resources used extensively by humans (i.e. see chapter about M. Lemire and A. Boyd).

Community perceptions are not always congruent with ecological concerns. Outrage, blame, and punishment (Sandman, 1987; Sandman et al., 1993) may be more important than the damage to the ecosystem itself. Further, in some cases, feelings of being pushed from their lands, or kept from their traditional hunting, fishing, and cultural lands influences perceptions of ecological resources (Flood & McAvoy, 2007). Importantly, it should be noted that communications about potential effects on species and ecosystems are going to be viewed through the eyes of the recipients, both in terms of how the message is crafted, and in what species or habitats the recipient is interested in. Different communities will be differentially interested in information about risk to ecosystems, have different perspectives on what endpoints in the ecosystem are important, and may be interested in different spatial or temporal scales of effects and recovery (Cappuyns, 2016). Environmental justice is only one of the cultural concerns (Bullard, 1990; Burger & Gochfeld, 2011; Chakraborty et al, 2016; EPA, 2019a,b; Gochfeld & Burger, 2011; Holifield, 2001). The responsibility of the ecologist is to provide accurate, informative information about the resources, and to recognize that values and relationships of people to the resources is an important ecological consideration (CDC 2020, DOE, 2020a; Burger et al., 2022).

## 3 | HANFORD SITE AS A CASE STUDY: MULTIPLE ECOLOGICIES, STAKEHOLDERS, AND MESSAGES

### 3.1 | The communication challenge

While describing the information that might be helpful to risk communicators faced with an event that affects eco-receptors and ecosystems is important, it is equally important to illustrate how ecological information may help the communicator and the audience. I use examples for the Department of Energy’s (DOE) Hanford Site in the State of Washington to illustrate some of the challenges. The Hanford Site not only has important ecological species and shrub-steppe habitats on its lands, it is also adjacent to one of only two free-flowing sections of the Columbia River where some of the main salmon spawning areas are located. Any physical disruption or contamination of the river, salmon spawning areas, and young salmon are important to governmental agencies, and Northwestern peoples, including Native American Tribes. Potential exposure to chromium leaking into the pore water from the Hanford Site creates a communication challenge about salmon, about the ecosystem, and for people fishing the salmon.

### 3.2 | The context

Much of the DOE land on the biggest sites is designated as National Environmental Research Parks, and DOE sites have unique, rare, and important habitats and species (DOE, 1994a,b; Dale & Parr, 1998; Whicker et al., 2004). The DOE is committed to protection of human health and the environment, as well as to environmental justice (DOE, 2020a,b). A few of the major DOE sites, as well as other contaminated lands, are located where there are environmental justice communities, such as Native Americans.

Communicators from governmental agencies (federal, local, Tribal), companies, community organizations, and others need to recognize the holistic nature of any community or ecosystem. Many of the counties, especially in the western United States, have a wide range of Native American communities as well as Hispanic, Black and Brown, and low-income communities that are often at risk (EPA, 2019a; DOE, 2020a). Moreover, the community may have long-standing issues with governmental approaches to their communities, leading to suspicion and distrust. For example, when the DOE built its nuclear facilities at Hanford Site (Washington), Oak Ridge Reservation (Tennessee), and the Savannah River (South Carolina), they needed a steady supply of water for cooling reactors, in low population density areas. They evicted the farmers, Tribes, and other inhabitants, creating a legacy of bad feelings that lingered across generations. Many of the farmers, other community members, and Native Americans settled nearby, but were generally unaware of the nuclear and chemical hazards until decades later (Gephart, 2010). This created an environmental justice situation for low-income and largely minority communities nearby when they realized their potential exposures to chemicals and radioactivity. It also leads to multiple communication challenges because of the long history of secrecy and no information, followed by incomplete information, distrust about radionuclide and other chemical contaminants on their traditional tribal lands, and finally the loss of historic, cultural, or religious lands and values.

Two prominent issues for Hanford and its neighbors are chromium contamination of the Columbia River and the persistence of nuclear weapons grade material and nuclear waste on site. Many environmental issues around some DOE sites are viewed within the context of past injuries and injustices, which colors communication among different stakeholders. The communications from DOE often did not address these concerns, but rather maintain that the remaining chromium and nuclear stockpiles are not a “risk” to their physical health. But to a Native American, degradation, destruction, and impacts on salmon and the remaining radionuclides defiles their traditional grounds. Moreover, they cannot easily visit their ancient burial grounds. They cannot collect their traditional herbs for food or ceremonial purpose. They do not know if their children will be exposed to unhealthy radionuclides or chemicals when they can go back on the site (to exercise their Treaty rights). The neighboring Tribes are precluded from conducting their annual cycle of activities as they had done for 9000 years (Bohnee et al., 2011; Butler & O-Connor, 2004). Tribal risk assessments, for example, calculate exposure using historic fish consumption levels up to 620 g/day (Harper & Harris, 2008), which DOE and EPA risk assessors consider unrealistic (Gochfeld et al., 2015). DOE does not fully trust the tribal assessments and values (CRITFC, 2013; Harris & Harper, 1998, 2000).

It has become circular. DOE communicates that the risk is extremely low from traditional foods, but the tribal members insist they would eat “historic levels” if they knew the foods were safe and they had access to them. In turn, cleanup levels are often determined by risk assessments that consider current consumption rates. Partly there is a lack of trust about future land use, sustainability, and the protection of ecological resources on these lands (Burger, 2019).

### 3.3 | Salmon exposure to chromium as a crisis

In addition to fish consumption, one contentious issue in the Northwestern Unites States, as well as at Hanford Site, has been the decline in many salmon stocks, which is of interest to Native Americans, US federal and State governmental agencies, recreational and commercial fisherman, conservationists, and the public-at-large (CRITFC, 2013; NRC, 1996). Hexavalent chromium added to the cooling water of the nine Hanford Reactors was a source of soil and water contamination. The potential effect of chromium on spawning salmon, their eggs and fry in the Columbia River created a communication challenge from the early 2000 on. Salmon have been declining for some time, partly as a result of a hydroelectric system of dams (Dauble et al., 2003a,b), harvest limits (Hyun et al., 2012a), genetic stability, and the large-scale supplementation of populations with hatchery fish (Holsman et al., 2012; Kostow, 2012). Restoring salmon populations to the Columbia River is a national priority (NRC, 1996; Williams, 2006). However, the occasional exceedances of chromium levels in the Columbia River adjacent to the Hanford Site, in the region of known salmon spawning grounds created immediate concern that chromium was one cause of salmon declines. For DOE, the risk from the few exceedances was small, and it was assumed that any chromium reaching the river was quickly reduced by dilution—the Columbia River is wide and deep, and free flowing. However, the chromium contamination was viewed by Native Americans as part of the larger issue of contaminating their traditional lands. For Native Americans, salmon are not only a subsistence and tribal food, but salmon are intimately connected to their religious and cultural tradition (see We are Salmon People, CRITFC, 2013) and have been for over 9000 years (Butler & O-Connor, 2004).

DOE, Washington State regulators, Tribal governments and members, and the general public wanted to know that chromium was not contributing to salmon declines, and they wanted an outside evaluation. Ultimately, we at CRESP (Consortium for Risk Evaluation with Stakeholder Participation) were asked to examine the issue and provide a report. Our overall goal was to understand whether and how chromium contamination discharges into groundwater upwellings into the Columbia River might affect the life history and population levels of salmon in the river, and the potential implications for remediation and restoration. Several CRESP researchers had already been meeting with DOE and state regulators for some time on a variety of other ecological and land use issues, and we also had been visiting and listening to elders and members of the Tribes about future land use considerations, access, contamination levels, and the role of outside, independent academics. In this case, it was important that the same people visited with the Tribes and others to establish a relationship—“we” was C. W. Powers, M. Gochfeld, and me.

### 3.4 | An approach

**FIGURE 2:**
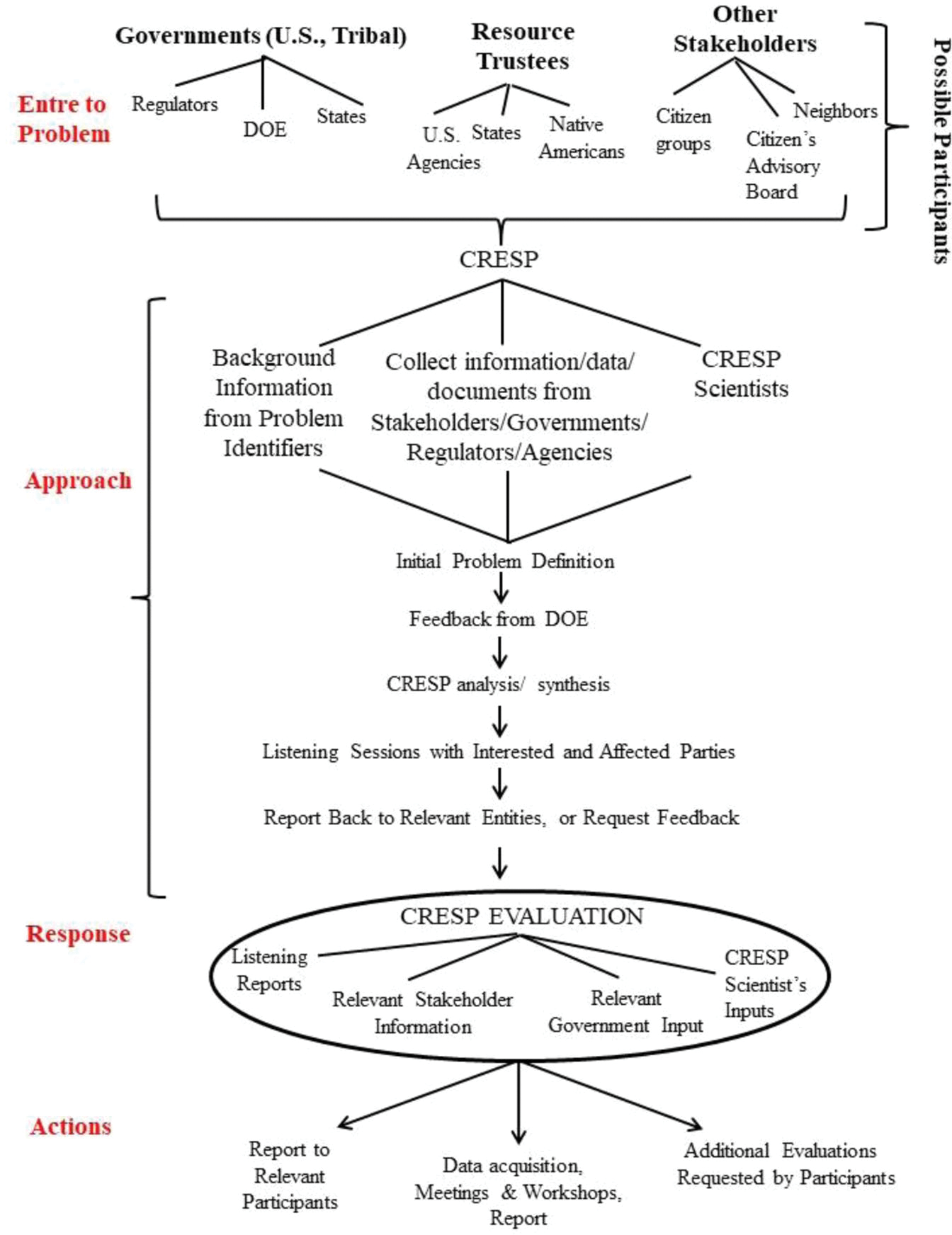
The general plan for listening, communication, and information acquisition to understand the potential risk to salmon in the Columbia River from chromium from the Hanford Site

When DOE asked CRESP to do an independent review of the potential effects of chromium on salmon, we visited state officials and tribal leaders several times during the development of the report. We met with the State representatives, who explained the state water quality criterion for chromium, and they suggested the usual academic sources of information for our studies (Figure 2). These meetings were frequent and consisted of mutual dialogs with questions and data presented. We also met with the Tribes in an informal manner as before. Often our “business” (communications about some actions, risks, or research) required less time than our “visiting” did. Such meetings led to mutual trust, empathy, and respect, allowing for more realistic risk communication. They shared their Tribal efforts to increase survival of salmon eggs and fry, and we visited their fish hatcheries well up the Snake River (Bohnee et al., 2011). We always had sufficient time to discuss how our families were, how our gardens were coming, how the salmon were running, and how the flowers and medicinal herbs were doing on the sun-parched hills we could see from their windows. We talked about our ancestors, and how each of our parents farmed the land. They took me to see where the best salmon were spawning, where the nearby dam was built by their parents and grandparents and described their wanderings on the Hanford Site (despite its being “offlimits”).

We participated in many listening sessions with Tribal elders and leaders in which we also learned of their knowledge of salmon spawning behavior, spawning sites and timing, potential effects of chromium on different life history stages, and visited their hatcheries. The Yakama Nation was particularly interested in the Boldt Decision (in 1974) that affirmed Tribal rights to fishing in the “usual and accustomed” places, in maintaining all the seasonal runs of salmon, and in the potential cellular and genetic effects of chromium on eggs and fry of salmon (R. Jim, November 2013). The Nez Perce knowledge was particularly useful because their focus was on increasing the survival of fry from their hatcheries, and they were conducting experiments on the resulting health of fry that experienced strong currents while developing (similar to natural conditions, but producing less viable fry than produced in “western-style hatcheries”), but in their view, “fry more able to survive natural conditions upon release,” (Bohnee, pers. comm, November 2013). The Yakama, Nez Perce, and Wanapum were all worried because the fry concentrate in shallow waters, where contamination might also concentrate (e.g., less dilution), causing adverse effects. They also questioned whether the fry would concentrate near warmer water in the winter, and whether this created an increased risk of chromium exposure. Other concerns were more basic –

“We catch salmon for a specific purpose for our culture and belief. We are worried (about radionuclides and chromium), yes, but we need to eat it because it is who we are.” (Rex Buck, Wanapum, Nov 11, 2013).

While we analyzed the literature and data, we continued to visit the Tribes and relevant stakeholders (Figure 2). We searched the literature for levels of chromium in salmon and for toxic levels of chromium in fish, as well as DOE databases for levels of chromium in the sediment and water of the Columbia River near Hanford (Eisler, 1986; EPA, 1986, 2009). The ecological information needed included ecology, behavior, and life cycle of salmon, habitats use for nesting by salmon, behavior of fry, river flow and potential effects of storms and river water level, pathway for exposure of salmon at different life cycles, contaminants in pore water (DOE, 2011a,b,c), and what specific characteristics of salmon were important to stakeholders, including for fishing, cultural, religious and existence values, among others (Bohnee et al., 2011; CRITFC, 2013; NRC, 1996).

### 3.5 | The outcome and communication responses

The tangible outcome of the project, which required over 5 years to complete, was a report to DOE laying out our findings (Burger et al., 2015; CRESP, 2015; Gochfeld & Burger, 2014). The main findings were that: (1) salmon life cycles are varied and complex (involving spring and fall runs, Healey, 1991), (2) having good population estimates by species and runs is critical (Hyun et al., 2012a,b), (3) several species of salmon spend a significant part of their life cycle in the Columbia River (and mainly in the Hanford Reach) where they spawn (Hayes et al., 2013), (4) contaminants are not viewed generally by the State or DOE as a significant stressor on salmon, especially compared to other stressors (CRESP, 2015; NRC, 1996; Regetz, 2003; Tiller et al., 2004), (5) laboratory experiments indicated that there is a great deal of variability in results, no effects were found at Hanford-relevant chromium exposures on fertilization, hatching, and exposure of alevins (early stage on salmon), but there were some laboratory effects of chromium on later stages of fry (Farag et al., 2006a,b; Patton et al., 2007), and (6) at some periods of time the fry may be vulnerable to chromium if they remain near the gravel surface where the pore water and river water meet (Burger et al., 2015; CRESP, 2015; Gochfeld & Burger, 2014). The Tribes, however, remained concerned about potential long-term effects of contaminants on eggs and fry of salmon (especially the DNA, R. Jim, Nov. 2014).

The process of communication and involvement over a long period of time resulted in acceptance by all parties of the report. It also resulted in inclusion of both “western” and “tribal” science and perspectives, as well as discussion of their more traditional, beliefs, current Washington state ambient surface water criteria for chronic exposure of hexavalent chromium, and EPA’s ambient water quality criterion. Communication was critical to the process, and knowledge of the process (and findings) was critical to communication. Further descriptions of the lessons learned are described below.

The final report was accepted because of the continued involvement of all parties (e.g., Figure 2). The report was the stated outcome for the project, but the process of communication itself was an equally important component. On-going discussions, negotiations, and agreements between all parties resulted in identification of the chromium source, and ultimate soil removal of potential future risks.

## 4 | LESSONS ILLUSTRATED AND LEARNED FOR SUCCESSFUL COMMUNICATION

There are many lessons learned about this essentially ecological and eco-toxicological issue—are salmon adversely affected by chromium, and by extension—are people and their cultures harmed? While the “science” for the answer existed in local Tribal information, published information on salmon populations and spawning behavior, published data on laboratory experiments with different levels of chromium on different life stages of salmon, and DOE data on chromium levels in pore water (that enters the Columbia River), the project was mainly one of information and communication among a range of different stakeholders. The lessons I learned will be described briefly below.

### 4.1 | Cultural history plays a role in how people view the risk to eco-receptors

For Northwestern peoples in general, salmon plays an important role in their culture and aesthetics, recreation, and commerce (NRC, 1996). The declines in most of the salmon stocks are also a result of economic development (e.g., hydroelectric dams, water control), recreational and commercial opportunities (fishing), and farming. For Native Americans, however, salmon are critical (CRITFC, 2013). The three federally recognized (and one state- recognized) tribes residing around the Hanford Site in the state of Washington still retain Treaty rights to conduct their traditional activities on site. Although, they have been largely prevented from access for the last 75 years, “cleanup” will open up more of the landscape to increased use of their traditional Treaty lands (hunting, fishing, gathering traditional foods and medicines, visiting burial grounds). Risk communication to these Native Americans has often failed because DOE and tribal risk assessors and risk communicators are working within different frameworks of exposure and of health risks from radionuclides (Harris & Harper, 1998). DOE meets infrequently with local tribes at their tribal offices, where they treat them as sovereign Nations as required by their US Treaties. Within this framework, there is no potential to shift to communication about the social and economic stresses the four Tribes face from the knowledge that some contaminants remain. This social and emotional toll was felt for decades when they were denied access to their traditional grounds, and when there was little communication with their elders or Tribal members. For many Native Americans, it is not just a matter of the existence of chemicals and radionuclide contamination on their Tribal Treaty lands, but that infrastructures and the destruction or degradation of ecosystems damages burial and hunting grounds, destroys their traditional medicinal and religious plants, and degrades viewsheds (Bohnee et al., 2011; Burger, 2019; Gochfeld et al., 2015; Landeen & Pinkham, 1999). Their culture and values require intact ecosystems, per se (Burger et al., 2010; Harris & Harper, 2000), and in their view the Columbia River was impacted, in this case, by chromium. The lack of understanding between the largely White DOE authorities and the Native Americans was partly a result of miscommunication and misunderstanding of the importance of the land itself, rather than only the role of DOE in reducing the risk from chromium contamination.

As we made more and more visits to the tribes, we learned a great deal about the importance of belief systems as well as absorbing some of their knowledge of the local ecosystems. In our case, we were familiar visitors, they liked being able to talk about the seasonal changes in plants and animals on the surrounding hills, and they appreciated us acknowledging their superior knowledge of the local life cycle and habitat choices of the salmon. Their knowledge focused our attention to the seeps in the shallows, where the young fry concentrated.

### 4.2 | Familiarity and previous mutual respect is essential

We were also a known entity to DOE, and Oregon and Washington State regulators and trustees; communication was easy and noncontentious. Similarly, the contractors and other publics engaged in a scientific and normal dialog with us about the risk of chromium for salmon eggs and fry. In the case of the Tribes, we were not “strangers” come to deliver a report, but known, empathetic people who they had trusted for information in the past. The Native American Tribes, as well as the DOE and state trustees and regulators, knew what we were doing at every step. We had talked to people during the literature searches, data analysis, and writing phase. Transparency, trust, empathy, and past shared experiences influenced how they viewed the results of our investigation. While we often arrived at Tribal offices with powerpoints, they were printed out, and we sat together and went over the studies we used to arrive at our conclusions. This worked because everyone there could ask questions, write notes on their copies, and provide us with additional information to include, or issues to explore. They could then show these to others in the Tribe. Personal interactions in small groups worked best with the Tribes, while power point presentations worked with larger groups.

Familiarity, mutual respect, and listening takes time, and in the present case it took over 5 years, but the end results are less contentious and far more acceptable to all parties. An audience is not homogeneous. Individual knowledge, values, and beliefs form mental models of how things started, how they progress, and what might evolve. Failure to recognize different mental models may interfere with risk communication (Bostrom et al., 2001). Thought not the topic of this paper, understanding and appreciating the mental models of all participants in any communication issue or crisis is critical.

### 4.3 | Inclusion is critical for communication

The issue of whether chromium was affecting populations of salmon required inclusion of a wide range of Tribal and US government agencies, as well as the public, other scientists, and other Tribal scientists. But it also, and perhaps more importantly, involved inclusion of the “science” of different groups of people. In the case of chromium and salmon populations, the knowledge of the Native American Tribes was critical: the Yakama provided detailed information of the locations of spawning, nests, and fry of the salmon, the Nez Perce provided information on the behavior of the fry and of experimental design for understanding risk to fry during development, and the Wanapum provided information on the nests, eggs, and behavior of the spawning salmon near their stretch of the Columbia River. All of our meetings provided additional sources of information and literature we might not have otherwise found. These aspects figured prominently in our analysis and report, and made the results credible and acceptable.

This background was important for interpreting the results of the few well-designed and well-controlled laboratory studies of chromium effects on various life stages of the salmon. Additionally, inclusion of related issues was an important communication component. While the Tribes were interested in chromium contamination in fish, they were also interested in whether there was contamination from the recent Fukushima accident that might adversely affect the fish, their spawning behavior, and their populations (Wada et al., 2013). This illustrates that for many communities, a one chemical approach may not be all that is needed. Data on effects from other accidents or exposures are relevant, and a risk communicator needs to know how, or from whom, to find this information.

### 4.4 | Ongoing chronic exposures can become communication crises

Exposure of biota (salmon in the case of Hanford) to radionuclides and chemicals is an on-going issue around most contaminated sites that requires continued evaluation of the ecological conditions around the biota, population status of biota, and population trends of biota. It becomes a crisis either when there is a new exposure (e.g., a spill or accident) to a given contaminant, or when managers, scientists or the public recognizes that a given chemical may be severely impacting populations. The crisis calls for communication of known scientific information, additional scientific information, synthesis of information, or just plain acknowledging that you do not know, or science does not know. Transparency for all these issues is critical. Further, knowledge increases over time with more observations, experimentation, and synthesis.

Also, times change and change the way people perceive, understand, and value the natural world. The unbridled developmental “wild-west” mentality of the late 1900s has been modulated by concerns over climate, global resilience, and the healthful benefits of green space and green activities (Condo et al., 2018). Stuart Udall captures concisely this in writing about the never-built Tocks Island Dam on the Delaware River. Over the course of 40 years the value of a “wild and scenic river” superseded the previous economic valuation of hydroelectric power, recreation, and flood control (Udall, 1987). Dams everywhere are under scrutiny in this new millennium (NRC, 1996).

### 4.5 | Looking and feeling like me

One of the elephants in the room for many communication crisis, whether human or environmental, is the lack of communicators that look like the intended concerned participants or audiences (Davis & Ramirez-Andreotta, 2021). While I am not a Native American, there are other commonalities that can contribute to trust and understanding. Growing up on a farm gave me a perspective of parents who could not always do what they wished with their land (“government people” had declared it wetlands even though his father had farmed it for decades), could not burn trash (because neighbors who had recently moved in did not like the smell), could not use some pesticides because neighbors did not like to see it, and could not protect crops from birds that ate them (because “laws” protected them). A farm upbringing also left me with a “feeling of reverence” for the land and its central and precarious role in life—a similar feeling to Native Americans. Although at Hanford, the Tribes had a 9000-year cultural history with the land, our own experiences with tradition and the land can serve as a bridge. Commonalities can be developed, but it takes time, exploration, and listening to the concerns and culture of others involved in the crisis. We each need to find our commonalities about our diversity so that we can interact equitable.

## 5 | CONCLUSIONS

Although in the Hanford case study I have concentrated on interactions with the four Native American Tribes, it is important to remember that communication was essential to the DOE, state and federal regulators, and natural resource managers, as well as to the general public through the Hanford advisory board. While the information and data were the same, the method of presentation and interactions varied, as did the time different stakeholders allotted to these discussions. Honesty, transparency, openness, and identifying data gaps were all essential for all groups, as was attribution to scientific sources and experiments. Not everyone’s objectives were satisfied, but each was considered, resulting in the final report being accepted when it was completed. Not everyone views the potential short-term effects of chromium on salmon as the key issue, and a recognition that cultural knowledge, scientific approaches, and even knowledge may differ is essential. In the end, the main sources of chromium contamination were eliminated by removing soil as a primary source of input into the pore water of the Columbia River.

One of the main lessons learned from the experience is that examining a question as important to so many different entities and stakeholders requires considerable time devoted to making sure that the science, approaches, and cultural values of everyone are considered. Time and persistence are important, but recognizing the different ways of listening, communicating, and understanding the science and the potential impacts of a chemical exposure are critical. Moreover, the time frame for many western approaches to science, remediation, and restoration extends to years or sometimes decades, while the time frame of Native American Tribes with Treaty Rights to the Hanford Site extends for centuries from 9000 years ago to the seventh generation into the future.

## Figures and Tables

**TABLE 1:** Major categories of information needed for crisis events with examples of public questions and concerns (after Burger et al., 2010, 2020)

Ecological information challenge(Key endpoints)	Ecological questions and concerns	Communication challenges and information needs

Species at risk	Endangered and threatened species (concern for all listed species)	What species are threatened and endangered; what might be at risk ecologically; and what do the different human community groups most care about? What is legally protected, and by what regulatory agency and interest group?
	Species of special management or conservation concern	Are there other species federal or state agencies are currently managing, are there species local people are concerned about? Are there barriers to the exposure and stressor, or can be built?
	Unlisted but rare species	Are there unlisted species being considered by agencies or the public to be listed (candidate species)? Are management or conservation projects vulnerable or impacted?
	Species of sport or commercial interests	What are the local wildlife species that are fished or hunted? Are they at risk? May some species benefit? Are aesthetic resources impacted?
	Species of cultural/religious concern	What are the local cultural or religious groups that use the region, what ecological resources do people care about? And what is most critical? What role do they play?
Species groups at risk	Habitats for groups of concern such as vernal ponds for frogs, interior forests for neotropical migrant birds	For any of the species at risk (from above list), what specific habitats might be most at risk, and is there anything to do about it, given the stressor?
	Suite of fish or wildlife for consumption	How can specific plants or wildlife that are consumed by people be protected? When will it be safe to consume them again?
Populations and communities at risk	For all the eco-receptor populations listed above	What are the dependencies among species that need protecting (e.g., food or prey for species at risk; possibility of increased predators)?
Unique habitats at risk	What habitats occur that are unique or rare for the region, area, and country	What habitats are ecologically important, and what ones do the local population care about. When can people go back onto a site? What landscape features affect habitat viability (e.g., patch size, connectivity, invasive species)?
	How are habitats impacted?Can/will they recover?	What will be washed away or destroyed? Covered or contaminated (by mud/sludge/toxics)/ Will time heal? Are dollars needed?
Eco-cultural resources	Any resource can be important to particular groups or cultures	What are these resources, which are most at risk, and how can they be made whole? Who are the community users of these resources? When can EJ and other communities use them again?
Temporal scales	How can the damage be reduced short term and long-term	How long will the species or habitats be affected? Provide assurance of the methods of limiting the scope of injuries, and making the ecosystem whole.
Spatial scales	How can the damage be limited in scope.	What are the methods used to reduce the spatial extent of the accident, spill or other stressor? Are there any natural barriers to the spread (e.g. a river may stop spread of fire). How can the region be restored?
Legal issues	Are there any state or federal laws or regulations related to eco-receptors on site or to habitats	What are the species that are listed on federal and state endangered species lists? Do you have the most recent information or lists (species status changes over time). Are there designated habitats of legal concern? Is a river designated “Wild and Scenic?”

**TABLE 2:** Details of some characteristics of interest to ecologists evaluating risk to ecosystems, and characteristics used to determine the efficacy of restoration. The characteristics (endpoints) on the right are a list that relate to the ecological level on the left (and are not in any order)

Ecological level	Characteristic or ecological endpoint for assessment

Individual and species attributes	Species populations versus individual considerations Does it matter in the long run if a few birds or fish die?
	Life stages
	Lifestyle (plant, herbivore, carnivore, omnivore)
	Media use (animals using water and/or land)
	Demographics (age distribution, gender)
	Reproductive potential (= recovery potential)
	Life span (and factors affecting life span, such as gender, environment)
	Individual and species susceptibilities
Community and ecosystem structure and function	Community processes (competition, predation, cooperation, coevolution)
	Relationship among different species (numbers of each)
	Structure of ecosystems (how much vertical structure is there?)
	Productivity (energy transfer)
	Food webs and pyramids are complex compared with simple food chains
	Energy and nutrient flow
	Disease and contaminant rates among species and communities
Landscape and regional considerations	Landscape matrix and spatial scale
	Patch size and interspersion
	Corridors (both aquatic and terrestrial)
	Refuges
	Relative percent of each type of habitat
	Built-out (how much ecological habitat will be available if all privately owned land is developed)
	Critical and unique habitats
	Are there offsets, compensation, NRDA, habitat “banks”
Global scales	All of the above in terms of global change
	Climate change and sea level rise effects (differentials by species, communities, and landscapes)
